# Parent-focused treatment for adolescent anorexia nervosa: a study protocol of a randomised controlled trial

**DOI:** 10.1186/1471-244X-14-105

**Published:** 2014-04-08

**Authors:** Elizabeth K Hughes, Daniel Le Grange, Andrew Court, Michele SM Yeo, Stephanie Campbell, Erica Allan, Ross D Crosby, Katharine L Loeb, Susan M Sawyer

**Affiliations:** 1 Department of Paediatrics, The University of Melbourne, 50 Flemington Road, Parkville, Melbourne, VIC 3052, Australia; 2 Centre for Adolescent Health, Royal Children’s Hospital, 50 Flemington Road, Parkville, Melbourne, VIC 3052, Australia; 3 Murdoch Childrens Research Institute, Melbourne, Australia; 4 Psychiatry & Behavioral Neuroscience, The University of Chicago, 5841 S. Maryland Ave., MC3077 Chicago, IL 60637, USA; 5 Neuropsychiatric Research Institute and University of North Dakota School of Medicine and Health Sciences, 700 First Avenue South, Fargo 57103, North Dakota, USA; 6 Fairleigh Dickinson University, 1000 River Road T-WH1-01, Teaneck, Hackensack, NJ 07666, USA

**Keywords:** Anorexia nervosa, Eating disorders, Adolescents, Family therapy, Randomised controlled trial

## Abstract

**Background:**

Family-based treatment is an efficacious outpatient intervention for medically stable adolescents with anorexia nervosa. Previous research suggests family-based treatment may be more effective for some families when parents and adolescents attend separate therapy sessions compared to conjoint sessions. Our service developed a novel separated model of family-based treatment, parent-focused treatment, and is undertaking a randomised controlled trial to compare parent-focused treatment to conjoint family-based treatment.

**Methods/Design:**

This randomised controlled trial will recruit 100 adolescents aged 12–18 years with DSM-IV anorexia nervosa or eating disorder not otherwise specified (anorexia nervosa type). The trial commenced in 2010 and is expected to be completed in 2015. Participants are recruited from the Royal Children’s Hospital Eating Disorders Program, Melbourne, Australia. Following a multidisciplinary intake assessment, eligible families who provide written informed consent are randomly allocated to either parent-focused treatment or conjoint family-based treatment. In parent-focused treatment, the adolescent sees a clinical nurse consultant and the parents see a trained mental health clinician. In conjoint family-based treatment, the whole family attends sessions with the mental health clinician. Both groups receive 18 treatment sessions over 6 months and regular medical monitoring by a paediatrician. The primary outcome is remission at end of treatment and 6 and 12 month follow up, with remission defined as being ≥ 95% expected body weight and having an eating disorder symptom score within one standard deviation of community norms. The secondary outcomes include partial remission and changes in eating pathology, depressive symptoms and self-esteem. Moderating and mediating factors will also be explored.

**Discussion:**

This will be first randomised controlled trial of a parent-focused model of family-based treatment of adolescent anorexia nervosa. If found to be efficacious, parent-focused treatment will offer an alternative approach for clinicians who treat adolescents with anorexia nervosa.

**Trial registration:**

Australian and New Zealand Clinical Trials Registry ACTRN12610000216011.

## Background

Anorexia nervosa (AN) is an eating disorder characterised by restricted dietary intake leading to low body weight, a fear of weight gain, and disturbed body image [1]. The highest incidence of AN is during adolescence [2], with 1.1% of adolescents having a lifetime history of AN or subthreshold AN (i.e., eating disorder not elsewhere classified; DSM-IV EDNOS) [3]. AN has a significantly detrimental impact on physical and psychological functioning, including having the highest mortality rate of all psychiatric conditions [2]. Current evidence supports family-based treatment (FBT) as an efficacious outpatient intervention for medically stable adolescents with AN [4-8]. Our research compares a parent-focused model of FBT in which parents and adolescents attend treatment sessions separately to a standard conjoint model.

FBT is a manualised outpatient treatment in which a mental health clinician assists parents to actively support weight gain and normalise eating patterns for their adolescent [9]. Treatment initially focuses on weight restoration. Parents are required to take control of meals, support their adolescent to eat, and prevent compensatory behaviours. Once weight is restored and resistance lowered, control of eating is returned to the adolescent and developmental issues can be addressed. Traditionally, FBT is delivered in a conjoint model whereby the whole family is seen together by the therapist (hereafter referred to as conjoint family-based treatment; CFT). While CFT allows the therapist to directly observe and intervene in family interactions, it also raises many challenges. For example, the content of sessions may not be suitable for all family members at all times, and some parents may be critical of their adolescent or display distress which impacts negatively on their adolescent [6,10,11]. The adolescent’s illness may also manifest in behaviours which interfere with the therapeutic process between the parents and therapist. In addition, there are practical issues that may prevent family members attending sessions together (e.g., work, school, travel) or that impede therapists being able to accommodate whole families (e.g., small office space) [12]. The CFT model also poses a challenge for dissemination of FBT, in that therapists without formal training in general family therapy are at times reluctant to treat within a conjoint format that includes patient, parents, other caregivers, and siblings. Of particular concern is the second session of FBT, an in-vivo family meal in which the therapist must facilitate parental efforts in the nutritional rehabilitation of their ill child, while mobilising the siblings to take on a supportive role. The success of this powerful session is one hypothesised mechanism of FBT, but no dismantling studies demonstrating its unique contribution to treatment outcomes have been conducted.

Separated models of FBT in which the adolescent and parents are seen in separate individual sessions can overcome many of the difficulties of CFT, may be just as effective as CFT, and may even be better suited to some families. In the earliest randomised controlled trial of separated family therapy (SFT) [5], 18 adolescents with AN received either CFT or a SFT model in which the therapist spent part of the session with the adolescent alone and part of the session with the parents alone. Both groups had a significant increase in weight, but although there was a somewhat greater increase in the SFT group (+19.9% expected body weight, EBW) compared to the CFT group (+13.2% EBW), group differences were not significant. In a subsequent larger trial, 40 adolescents with AN were randomised to CFT or SFT [6]. Using Morgan-Russell outcome criteria [13], 76% of the SFT group had good or intermediate outcome at end of treatment compared to 47% of the CFT group, with outcomes improving further at 5-years follow-up (SFT = 90% and CFT = 78% good/intermediate outcome) [11]. Once again, however, the group differences were not statistically significant. Of importance, in both trials there was evidence that adolescents from critical families (i.e., those with high expressed emotion) had poorer outcome in CFT than in SFT [5,6,11].

While the sample size of these studies limits the interpretation of their findings, they provide preliminary indications that SFT produces similar, and potentially superior, outcomes to CFT. They further suggest that, at least for some families, SFT may be uniquely clinically indicated. For the current study, we amplified the differences between CFT and SFT to develop a separated model of treatment in which the therapist sees only the parents in treatment sessions while a nurse attends to the monitoring of the adolescent’s weight, mental and medical status and plays a supportive role. By extension, there is no family meal session in this model. Unlike CFT and previous separated models of FBT, in this treatment parents are for the most part the exclusive participants in therapy, similar to other parent training-based psychological interventions [14]. Hence, the therapy was named parent-focused treatment (PFT). The structure of PFT is described in the Methods section.

### Aims and hypotheses

The aims of this study are to:

1. Examine the relative benefits of PFT compared to CFT in a randomised controlled trial with a cohort of outpatients with AN and EDNOS- Anorexia Nervosa type (EDNOS-AN);

2. Explore predictors and moderators of treatment response; and

3. Explore mediators of treatment response.

It is hypothesised that:

1. PFT will be more effective than CFT as indicated by more participants achieving full remission and partial remission after 6 months of treatment and at 6 and 12 months follow-up;

2. Adolescents with parents who are highly critical and are assigned to PFT will have better outcomes compared to adolescents with parents who are highly critical and are assigned to CFT;

3. Regardless of treatment assignment, outcomes will be poorer for adolescents with co-morbid psychiatric illness, high levels of obsessionality, features of personality disturbance, parent psychopathology, high levels of parental criticism, poor therapeutic relationship, and/or low expectation for treatment effectiveness. These factors will also be explored as potential moderators of outcomes with respect to treatment assignment;

4. Treatment response will be mediated by reduced dietary restraint and depressive symptomatology, and increased self-esteem, parent efficacy and family functioning.

## Methods

### Overall study design

**Figure 1:**
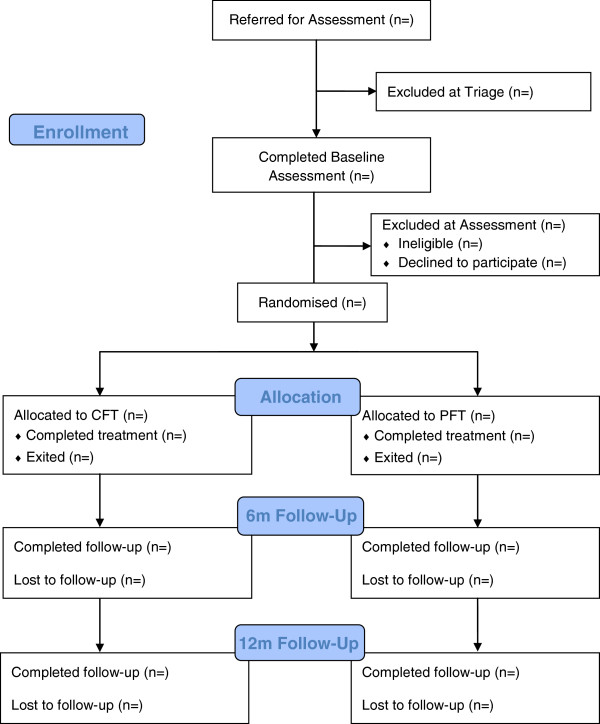
Participant flowchart.

The study is a randomised controlled trial (RCT) of two active interventions: CFT and PFT. Each participant receives 6 months of treatment and is followed up to 12 months post-treatment completion. Participant flow through the study is shown in Figure 1. Study recruitment began in July 2010 and is estimated to continue until July 2014, with the final 12-month follow-up assessment due to be completed in December 2015. The study is registered with the Australian and New Zealand Clinical Trials Registry (ACTRN 12610000216011). Ethics approval has been granted by the Royal Children’s Hospital Human Research Ethics Committee (#30035).

### Setting

The Royal Children’s Hospital Eating Disorder Program is a multidisciplinary specialist program that provides inpatient and outpatient medical management and outpatient FBT for adolescents with AN (see [15] for a detailed program description). The team includes clinicians from the disciplines of paediatrics, psychology, psychiatry, nursing, dietetics, and social work. Patients are referred by their general practitioner or other provider. Following a standardised phone triage conducted with the parents by a clinical nurse consultant, the patient and their parents attend a one-day multidisciplinary assessment clinic for diagnostic evaluation, medical examination and treatment planning. This assessment includes administration of standardised assessments by research staff not involved in treatment delivery. Prior to attending the assessment clinic, families are sent an information pack including written questionnaires.

Patients are also referred for outpatient FBT by the Royal Children’s Hospital adolescent inpatient unit. These patients and their parents undergo the same assessment while an inpatient and commence treatment upon discharge.

### Participants

Participants will be 100 families of adolescents aged between 12 and 18 years inclusive. Eligible participants are identified during triage and intake assessment. Sample size calculation for this study was based upon two controlled trials of FBT [6,8]. It was calculated that for 80% power and a two-tailed significance level of .05, it would be possible to detect an 27% difference between CFT and PFT (dichotomous outcome of remitted versus non-remitted) with a sample size of 45 participants per group. Assuming a dropout rate of 10%, the enrolled sample size requires 50 participants per group.

Treatment groups will be compared on sociodemographic and clinical characteristics at baseline using Fisher’s Exact test for dichotomous variables (e.g., gender, family status), chi-square for categorical variables (e.g., ethnicity, comorbid diagnoses), and independent sample t-tests for continuous measures (e.g., age, % EBW).

### Inclusion criteria

Participants are eligible to participate if they are aged between 12 to 18 years, meet DSM-IV criteria for AN (excluding the amenorrhea criterion) or EDNOS-AN [16], are sufficiently medically stable for outpatient treatment, and are living with at least one parent or guardian who can read and speak English to at least a sixth-grade level. DSM-IV criteria were applied as the trial commenced prior to publication of the DSM-5 [1]. In anticipation of the DSM-5 criteria, however, the amenorrhea criterion was excluded and, in the absence of explicit acknowledgement of fear of weight gain or becoming fat, persistent behaviours that interfere with weight gain are considered diagnostic. Weight thresholds are ≤85% EBW for AN and 85-90% EBW for EDNOS-AN. For adolescents at or above the 75^th^ percentile for height, the weight threshold was set higher (≤95% EBW). EBW is calculated from median body mass index for age and gender using the Centers for Disease Control charts [17]. Medical stability is determined by a paediatrician or nurse according to a standard protocol that was informed by published guidelines [18].

### Exclusion criteria

Participants are excluded from entry into the study if they present with current psychotic disorder, dependence on drugs or alcohol, explicit suicidal risk (i.e., requiring hospitalisation or high level supervision), or a physical condition known to influence eating or weight (e.g., pregnancy, cancer). Participants who have previously received 10 or more FBT sessions with a trained FBT clinician are also excluded. Participants on psychotropic medication who meet inclusion criteria for the study are only eligible for participation if they have been on a stable dose of for a period of at least 8 weeks and the medication has not been prescribed for the primary purpose of weight gain. In the case of patients with current or a history of recent or past physical or sexual abuse by a family member, the perpetrator of the abuse is excluded from treatment. If physical or sexual abuse by a family member occurs in the course of treatment, the perpetrator is excluded from ongoing treatment and appropriate protective services are notified if indicated by Victorian laws.

### Recruitment

Information statements about the RCT are included in the information pack posted to each family prior to attending the assessment clinic. Following completion of the intake assessment and feedback regarding diagnosis, a researcher explains the treatment to the parents and patient and, if eligible, informs them of the RCT and invites them to participate. If the family agrees, the adolescent and parents sign written consent forms. Consent includes a choice of whether to have assessment and treatment sessions audio recorded and whether to allow data to be used for other future ethically-approved research.

### Randomisation

A statistician independent of the research group generated a randomisation schedule using block randomisation with variable block sizes (currently masked from research personnel). Randomisation is stratified by eating disorder severity (low vs. high) to ensure equal allocation to each of the treatment arms. High severity is defined as <80% EBW, illness duration >12 months, and the presence of one other psychiatric diagnosis (i.e., all three criteria must be met). The randomisation schedule is only accessible by designated staff members at the Royal Children’s Hospital who are independent of the Eating Disorders Program team including the research team. When randomisation is required, a researcher sends an email with the participant identification number and severity category to one of the independent staff members requesting randomisation. The allocation is sent via reply email and the researcher communicates the allocation to the family and treating clinicians.

### Interventions

All participants, regardless of treatment group, receive 18 therapy sessions over 6 months. The frequency of sessions is the same across treatment groups: twice weekly in weeks 1 and 2, weekly for weeks 3 to 10, fortnightly for weeks 11 through 18, and every third week for weeks 19 to 24. In other words, dose and intensity of treatment are the same for CFT and PFT.

*Conjoint Family-Based Treatment (CFT)*. In CFT, the whole family is expected to attend treatment sessions. This includes the adolescent, both parents (for two-parent families), and siblings. Treatment progresses through three phases detailed in the treatment manual [9]. In the first phase (sessions 1–12), therapy is focused on the eating disorder and includes a family meal undertaken in session two. This phase is characterised by attempts to absolve the parents from the responsibility of causing the disorder, and by capitalising on the strengths of their parenting to facilitate their offspring’s nutritional rehabilitation. Families are encouraged to work out for themselves how best to restore their child’s weight, drawing on a combination of common-sense practices (i.e., FBT asks parents, “What would you be feeding your child now if s/he were underweight for a medical reason?” and cautions them against symptom accommodation) and the eating disorders expertise of the therapist. In Phase 1, the majority of weight restoration is accomplished. In Phase 2 (sessions 13–16), once weight restoration is nearing completion, parents are helped to transition eating and weight control back to the adolescent in a developmentally appropriate manner. The third phase (sessions 17–18) is initiated when the patient achieves a stable weight and self-starvation has abated. The central theme is the establishment of a healthy adolescent stage of development in all aspects of functioning, including the patient’s relationship with his or her parents. In CFT, the mental health clinician weighs the patient at the beginning of each session and spends approximately 10 minutes with the adolescent before bringing the rest of the family in for the remainder of the 50-minute session.

*Parent-Focused Treatment (PFT).* In PFT, treatment progresses through the same three phases as CFT [9], however the parents and adolescent are seen in separate sessions. Each appointment begins with the adolescent being seen by the clinical nurse consultant who weighs the adolescent, assesses medical stability as needed, and provides brief supportive counselling for up to 15 minutes. The nurse communicates the weight and any other pertinent information to the therapist who then sees the parents for 50 minutes. The focus and content of the parent sessions are the same as in CFT, but without direct interaction with the adolescent. The only direct contact between the therapist and adolescent is at the first session, when the therapist briefly introduces themselves, and at the end of the final session, when the therapist farewells the family. As noted above, there is no family meal in PFT. Siblings are not included in PFT treatment sessions but other caregivers involved in weight restoration can be included (e.g., grandparent). A study treatment manual was developed for PFT and a detailed description of PFT will soon be published [19].

### Treatment fidelity

Therapists participate in weekly clinical supervision either in-person or via teleconference with an expert FBT clinician (authors DLG and KLL) to review treatment progress and ensure adherence to the treatment protocol. All treatment sessions for which consent has been provided are audio recorded. Randomly selected recordings are reviewed by author DLG throughout the trial.

### Additional patient monitoring

Medical oversight is provided at an outpatient appointment with a paediatrician at a minimum of every five weeks. The decision to hospitalise patients is made by the paediatrician and clinical team according to the team’s eating disorders admission protocol. If a participant requires hospitalisation for ≤ 21 days during the treatment phase of the study, they return to their allocated therapeutic arm after discharge and complete the treatment course. Admissions to hospital longer than 21 consecutive days or a third admission during treatment results in exclusion from the study treatment phase. Such participants are offered continued treatment as recommended by the clinical team.

Referrals can be made to the team psychiatrist for assessment of mental status and management of co-morbid mental health conditions and psychiatric crises as required. Once treatment has commenced, psychotropic medication can be prescribed by the psychiatrist for comorbid conditions (e.g., depression, anxiety). If the condition is not pre-existing, the EDE [20] is administered prior to commencement of medication.

### Measures

The study assessment timeline is shown in Table 1. The assessments are part of routine care of all patients in FBT at the service. Face-to-face assessments are conducted at baseline, week 4, week 12, end of treatment (i.e., Week 24), and at 6 and 12 months post-treatment completion. Written questionnaires are completed at each of these face-to-face assessments and at fortnightly intervals during the first 12 weeks of treatment. As a token of appreciation and reimbursement for time and travel, parents and adolescents receive a $20 gift card for attending each assessment at mid-treatment, end-of-treatment and follow-up. Participants who complete all written questionnaires are entered into a yearly draw for a $100 gift card.

**Table 1:** Study measures and time points

**Measure**	**Source**	**Baseline**	**Each treatment session**	**Week 1**	**Week 2**	**Week 4**	**Weeks 6, 8 & 10**	**Week 12**	**Week 24 (End of Treatment)**	**6mth & 12mth FU**
Weight	Adolescent	✓	✓			✓		✓	✓	✓
Height	Adolescent	✓						✓	✓	✓
Menstrual status	Adolescent	✓	✓					✓	✓	✓
Binge/purge frequency	Adolescent	✓	✓			✓		✓	✓	✓
Eating Disorder Examination	Adolescent	✓						✓	✓	✓
	Parent	✓						✓	✓	✓
Mini International Neuropsychiatric Interview	Adolescent	✓							✓	✓
	Parent	✓							✓	✓
Children’s Yale-Brown Obsessive Compulsive Scale	Adolescent	✓				✓		✓	✓	✓
Yale-Brown-Cornell Eating Disorder Scale	Adolescent	✓				✓		✓	✓	✓
Five Minute Speech Sample	Parent	✓				✓		✓	✓	✓
Symptom Checklist-90-R	Parent	✓								
Family Environment Scale	Adolescent	✓			✓	✓	✓	✓	✓	✓
	Parent	✓			✓	✓	✓	✓	✓	✓
Borderline Personality Questionnaire	Adolescent	✓						✓	✓	✓
Children’s Depression Inventory	Adolescent	✓			✓	✓	✓	✓	✓	✓
Rosenberg Self-Esteem Scale	Adolescent	✓			✓	✓	✓	✓	✓	✓
Eating Disorders Examination-Questionnaire	Adolescent	✓			✓	✓	✓	✓	✓	✓
Positive and Negative Affect Scale	Adolescent	✓			✓	✓	✓	✓	✓	✓
	Parent	✓			✓	✓	✓	✓	✓	✓
Parents Versus Anorexia Scale	Parent	✓			✓	✓	✓	✓	✓	✓
Therapy Suitability and Patient Expectancy	Adolescent			✓	✓	✓	✓	✓	✓	
	Parent			✓	✓	✓	✓	✓	✓	
Helping Relationship Questionnaire	Adolescent				✓	✓	✓	✓	✓	
	Parent				✓	✓	✓	✓	✓	

*Eating Disorder Examination (EDE)*[21]. The EDE is a semi-structured interview used to diagnose eating disorders. It measures eating-related attitudes, cognitions, and behaviours primarily during the past 28 days. The EDE yields four subscale scores (Restraint, Eating Concern, Weight Concern, and Shape Concern) and a global score measuring the overall severity of eating disorder psychopathology. Behavioural symptoms of eating disorders (e.g., binge eating, purging) are assessed through a series of diagnostic questions about the frequency of behaviours in the 3–6 months prior to assessment. The EDE has good reliability and validity [22] and has been used in several studies of paediatric eating disorders [8,23].

A parent-report version (PEDE) is also utilised in this study [24,25]. Findings from the EDE and PEDE are integrated with the psychiatrist’s assessment to arrive at the diagnosis at baseline.

*Expected Body Weight (% EBW).* This is calculated as [current BMI]/[50^th^ percentile BMI] × 100. The 50^th^ percentile BMI is determined using the Centers for Disease Control charts relative to gender and age to the closest 6 months [17]. Weight is measured to the nearest 0.05 kg using regularly calibrated digital scales. A trained researcher weighs the adolescent in a gown, and the therapist/nurse weighs the adolescent in light indoor clothing without shoes. Height is measured to the nearest 0.1 cm using a wall mounted stadiometer. A trained researcher measures the adolescent’s height without shoes using a standard protocol.

*Binge/Purge Frequency.* Binge eating and purging episodes in the previous week are assessed by self-report at each treatment session and each assessment time point.

*Mini International Neuropsychiatric Interview for Children and Adolescents (MINI-Kid)*[26]. The MINI-Kid is a structured clinical interview for assessment of DSM-IV psychiatric disorders in children and adolescents. The MINI-Kid has been shown to be a reliable and valid assessment of psychiatric disorders [27,28]. The results of the MINI-Kid self-report and parent-report versions are integrated with the psychiatrist’s assessment to arrive at a final diagnosis at baseline.

*Children’s Yale-Brown Obsessive Compulsive Scale (CY-BOCS)*[29]. The CY-BOCS is a semi-structured interview designed to assess the presence and severity of obsessions and compulsions in children. The CY-BOCS was modified for children from the adult Yale-Brown Obsessive Compulsive Scale (Y-BOCS) [30]. The CY-BOCS has been shown to have high internal consistency as well as good convergent and divergent validity [29].

*Yale-Brown-Cornell Eating Disorder Scale (YBC-EDS)*[31]*.* Based on the Y-BOCS, the YBC-EDS is designed to assess the presence and severity of obsessions and compulsions related to eating behaviours, weight, and exercise. Studies of patients with eating disorders have demonstrated the YBC-EDS has good reliability and validity, and is sensitive to change over the course of therapy [31,32].

*Five Minute Speech Sample (FMSS)*[33]. The FMSS was developed as a brief measure of expressed emotion in family members of individuals with a psychiatric illness. The parent is asked to speak for 5 minutes about their child and the content is coded by trained assessors. Content is rated as either high or low in expressed emotion based on level of criticism and emotional over-involvement displayed during the speech sample. In studies of relatives of individuals with schizophrenia [33,34] and individuals with AN [35], the FMSS has been shown to be comparable to the Camberwell Family Interview [36], a 1–2 hour long semi-structured interview considered the gold standard for assessing expressed emotion.

*Symptom Checklist-90-R (SCL-90-R)*[37]*.* The SCL-90-R is a 90-item self-report measure of psychiatric symptomatology including depression, anxiety, obsessive-compulsive, and psychotic symptoms. The respondent indicates the degree to which they have been distressed by each symptom during the preceding week. Studies have shown the SCL-90-R to have good internal consistency and to be a valid measure of general psychiatric distress [38,39].

*Family Environment Scale (FES)*[40]*.* The 27-item relationship dimension of the FES is used to assess the degree of cohesion, expressiveness, and conflict in the family. This scale has been used in numerous studies to assess family functioning including studies of eating disorder symptomatology [41]. The FES has been shown to have good internal consistency, to correlate with other measures of family environment, and to change with family-focused interventions [40].

*Borderline Personality Questionnaire (BPQ)*[42]*.* The BPQ is an 80 item, self-report measure of borderline personality traits as defined by the DSM-IV (e.g. affective instability, impulsivity, self-harm, fear of abandonment, relationship difficulties, and identity issues). This questionnaire has been shown to have good internal consistency and validity [42,43]. In a sample of Australian adolescents, the BPQ was found to have high internal consistency, diagnostic accuracy, high test-retest reliability, and moderate sensitivity [44].

*Children’s Depression Inventory (CDI)*[45]. The CDI is a 27-item self-report measure of depressive symptomatology experienced by children and adolescents in the past 2 weeks. Studies have shown the CDI to have high internal consistency and to be a valid measure of overall level of depressive symptomatology in children and adolescents [46,47]. The suicidal ideation item is not included in this study.

*Rosenberg Self-Esteem Scale (RSE)*[48]*.* The RSE is a widely used self-report instrument of 10 items measuring an individual’s overall self-esteem. A meta-analysis of 23 factor analytic studies including 32,491 participants found support for a one-factor model of self-esteem as measured by the RSE [49].

*Eating Disorders Examination-Questionnaire (EDE-Q)*[50]*.* The EDE-Q is a self-report measure adapted from the EDE. Only the 5-item Dietary Restraint subscale is used in this study to assess attempts to restrict dietary intake. The EDE-Q Restraint Scale has high internal consistency [51] and results are highly comparable to those assessed using the EDE interview [52].

*Positive and Negative Affect Scale (PANAS)*[53,54]. The PANAS is a self-report measure of positive and negative affect. The respondent is presented with a list of feelings (e.g., sad, happy, irritable) and ask to indicate to what extent they felt that way in the past 2 weeks. It is a well validated, reliable and widely used measure of emotional experience for use with both adults and children [53,54]. The current study utilises 41 items from the PANAS Expanded Form [53] to derive Positive Affect and Negative Affect scales as well as the Fear, Hostility, Guilt, Sadness and Joviality subscales. Adolescents report on their own emotional experience and parents report on their observations of their child’s emotional expression.

*Parents Versus Anorexia Scale (PVA)*[55]. This is a 7-item measure of self-efficacy of parents in FBT; that is, parents’ perceptions of their ability to bring about recovery in their child [55]. The measure was designed to align with the principles of FBT outlined in the manual [9]. The PVA has been found to have acceptable internal consistency and to be positively correlated with total increase in % EBW during FBT [55].

*Therapy Suitability and Patient Expectancy*[56]*.* Parent and adolescent perception of the suitability of the treatment is assessed with the question “How suitable do you think this therapy is for your problem?” Their expectation of improvement with treatment is assessed with the question “How successful do you think therapy will be?” Each item is rated on an 11-point scale (0 = *Not at all*, 10 = *Extremely*). The questions were adapted from those used in a trial of FBT for bulimia nervosa [56].

*Helping Relationship Questionnaire (HRQ)*[57]. The HRQ measures two main aspects of the therapeutic relationship: the experience of being helped and supported, and the experience of being in a collaborative effort with the therapist. Eleven items are rated on a 6-point scale (-3 = *Strongly feel it’s true*, +3 *Strongly feels it’s untrue*). Two additional open-ended questions ask the respondent to comment on the ways they feel improved and the ways they feel worse, and a final item asks the respondent to rate their improvement so far (1 = *Not at all*, 5 = *Very much*). The HRQ has been used in a trial of FBT for bulimia nervosa [56].

### Primary outcome

The primary outcome is full remission, defined as ≥ 95% EBW and an EDE Global score within one standard deviation of community norms.

### Secondary outcomes

The secondary outcomes are partial remission (defined as ≥ 85% EBW) and changes in eating pathology (EDE subscales and behaviour frequency scores), depressive symptoms (CDI) and self-esteem (RSE).

### Data analysis

Remission will be determined separately at post-treatment and follow-up. The primary outcome analysis will be based upon intent-to-treat. In those cases in which there are missing post-treatment or follow-up data, the pre-treatment observation will be carried forward to characterise that participant’s response [58]. Groups will be compared on remission at post-treatment and follow-up using two-tailed Fisher’s Exact test with *alpha* set to .05. The secondary outcome category is the proportion of participants partially remitted. CFT and PFT will be compared at post-treatment and follow-up using Fisher’s Exact test.

A secondary analysis will be performed using a mixed effects linear regression model [59] to compare CFT and PFT participants on eating pathology, depression and self-esteem at mid-treatment (week 12), end-of-treatment (week 24), 6-month follow-up, and 12-month follow-up controlling for pre-treatment levels. Analyses will be performed on log-transformed variables for behavioural frequencies to satisfy the assumptions for the linear model. Random regression models allow for the inclusion of individuals with missing data. Consequently, no data imputation will be performed. Comparisons between groups will be based upon the main effect for group with a two-tailed alpha of .05. *Post hoc* comparisons between groups at specific time-points will be based upon a two-tailed Bonferroni corrected *Post hoc* comparisons between groups at specific time-points will be based upon a two-tailed Bonferroni corrected *alpha* of .0125 (.05/4).

Moderation analyses will be performed using logistic regression analyses, with models using main effects for treatment, moderator, and treatment-by-moderator analyses to predict remission at post-treatment and follow-up assessments. Bootstrap mediation analyses with bias-corrected confidence intervals will be performed using the *PROCESS* macro developed by Hayes [60].

## Discussion

This trial will be the largest randomised trial of a separated model of FBT for adolescents with AN to date. If found to be efficacious, PFT has the potential to increase recovery rates by offering clinicians and families an alternative approach to standard conjoint FBT.

Although standard FBT has the most promising evidence-base for treatment of adolescent AN [61,62], it has significant limitations. As described earlier, the standard conjoint format described in the manual (i.e., CFT) [9] raises many challenges and is difficult for many families and therapists to adhere to for a variety of reasons [12,19]. Moreover, studies using strict outcome criteria suggest CFT only achieves remission in around 50% of adolescents [6,8]. As individual therapies are generally found to be less effective in this population [8,63], clinicians and researchers tend to focus on improving outcomes by modifying FBT. This trial builds on two previous studies that indicated restructuring FBT with parents and adolescents attending separate sessions may be an efficacious alternative treatment for some families.

Of importance, the trial will not only indicate whether PFT is a more efficacious alternative to CFT, but moderator analyses will indicate whether there are specific patient or family characteristic that are useful in guiding the selection of treatment approach. For example, previous research suggests highly critical families may be better suited to SFT rather than CFT [5,6] and that adolescents with high eating-related obessionality are better suited to CFT than adolescent-focused therapy [64]. Furthermore, mediation analyses in this trial will indicate potential mechanisms of action which could be the target of future efforts to enhance FBT. For example, if remission is shown to be mediated by improved parent efficacy, techniques could be integrated into FBT to increase efficacy in parents who struggle with this during treatment.

So far the study has progressed smoothly and is expected to conclude in December 2015 as scheduled. Recruitment rates have been somewhat uneven, being higher than expected initially and in recent months, and lower between times. Periods of slowed recruitment have been largely attributable to families who present to the program being ineligible for the trial. Of note, 33% (70/214) of adolescents assessed between July 2010 and January 2014 were above the % EBW cut-off for the trial. Most of these were diagnosed with EDNOS-AN type, with just nineteen excluded for reasons other than weight (e.g., age, language, diagnosis). Our program has noted a rise in this type of presentation [65]. Defined in the DSM-5 as atypical anorexia nervosa, these adolescents have all the features of AN but despite significant weight loss are not underweight [1]. This appears to be an important emerging population that will require future research investment.

In conclusion, this trial will address an important clinical need by testing a modified model of FBT for adolescent AN in one of the largest randomised trial of its kind. AN is a life-threatening psychiatric illness which has a profound impact on an adolescent’s physical, psychological and social functioning [2]. The development of treatments that are effective, acceptable and achieve rapid remission during this critical developmental period is crucial. The trial with also provide an indication of factors which moderate and mediate treatment outcome, the results of which will be important to both the dissemination of PFT and for further refinement of this treatment and other forms of FBT.

## Abbreviations

AN: Anorexia nervosa; BPQ: Borderline personality questionnaire; CDI: Children’s depression Inventory; CFT: Conjoint family-based treatment; CY-BOCS: Children’s Yale-Brown Obsessive compulsive Scale; EBW: Expected body weight; EDE: Eating Disorder Examination; EDNOS: Eating disorder not elsewhere classified; FBT: Family-based treatment; FES: Family Environment Scale; HRQ: Helping Relationships Questionnaire; PANAS: Positive and Negative Affect Scale; PFT: Parent-focused treatment; RSE: Rosenberg Self-esteem Scale; SFT: Separated family-based treatment; YBC-EDS: Yale-Brown-Cornell Eating Disorder Scale.

## Competing interests

DLG receives royalties from Guilford Press and Routledge, and consultant fees from the Training Institute for Child and Adolescent Eating Disorders.

The remaining authors declare that they have no competing interests.

## Authors’ contributions

EKH, DLG and SMS contributed to the overall design and conception of the study. SMS was responsible for writing the grant application. EKH, DLG, AC, MSMY, SC, EA, KLL, and SMS contributed to the detailed study protocols, study implementation and coordination. RC was responsible for the statistical analytic plan including power analysis. EKH drafted this manuscript and all authors commented and revised this manuscript. All authors have read and approved the final manuscript.

## Pre-publication history

The pre-publication history for this paper can be accessed here:

http://www.biomedcentral.com/1471-244X/14/105/prepub
